# Trait Diversity of Pulse Species Predicts Agroecosystem Properties Trade-Offs

**DOI:** 10.3389/fpls.2021.636915

**Published:** 2021-03-31

**Authors:** Julie Guiguitant, Denis Vile, Hélène Marrou

**Affiliations:** ^1^SYSTEM, Montpellier SupAgro, INRAE, CIRAD, Institut Agronomique Méditerranéen de Montpellier, CIHEAM, University of Montpellier, Montpellier, France; ^2^LEPSE, Univ Montpellier, INRAE, Institut Agro, Montpellier, France; ^3^African Integrated Plant and Soil Research Group (AiPlaS), AgroBioSciences, Mohammed VI Polytechnic University, Ben Guerir, Morocco

**Keywords:** agroecosystem services, functional diversity, leaf nitrogen content, legumes, pulse crops, trait-based agroecology, traits–services trade-offs

## Abstract

Crop diversity management in agriculture is a fundamental principle of agroecology and a powerful way to promote resilient and sustainable production systems. Pulses are especially relevant for diversification issues. Yet, the specific diversity of legumes is poorly represented in most cropping systems. We used the trait-based approach to quantify the functional diversity of 30 pulses varieties, belonging to 10 species, grown under common field conditions. Our aim was to test relationships between traits, yield, and supporting agroecosystem properties. Our experimental results highlighted trade-offs between agroecosystem properties supported by different combinations of traits. Also, results demonstrated the relevance of leaf nitrogen content (LNC), leaf area ratio (LAR), and reproductive phenology to predict most of the trade-offs observed between agroecosystem properties. A comparison with a previous analysis based on literature data collected in diverse agronomic situations suggested that some traits are more plastic than others and therefore contribute differently to frame legumes diversity depending on the conditions of observation. Present results suggested that the implementation of such trait-based approach would rapidly benefit the selection of species/varieties for specific targeted agroecosystem services provisioning under specific (environmental or management) conditions.

## Introduction

Crop diversity management in agriculture is a fundamental principle of agroecology and a powerful way to promote resilient and sustainable production systems (Gaba et al., 2015). Agroecosystem management offers multiple alternatives to monoculture, such as diversification with pulses, through intercropping or rotation. Their well-known ability to fix atmospheric nitrogen reduces fossil energy consumption and makes them particularly suitable for low-input systems. They are also particularly important in human nutrition as a source of proteins to complement cereal-based diets (Singh et al., 2003; Tharanathan and Mahadevamma, 2003). Yet, the specific diversity of legumes is poorly represented in most cropping systems. While more than 80 pulses species are known to contribute to human diet, the FAO database includes only 11 of them (Tiwari et al., 2011). This lack of attention creates a risk of continued erosion of knowledge and genetic resources that could further hamper the integration of these crops into more diversified cropping systems (Dansi et al., 2012).

To increase diversity within cropping systems, choosing appropriate species is an important prerequisite (Brooker et al., 2015). The trait-based approach is well suited for designing diversified cropping systems capable of providing specific sets of services. In ecology, the approach has been increasingly employed to link biodiversity to ecosystem functioning and services (Lavorel and Garnier, 2002; Violle et al., 2007; Garnier and Navas, 2012); a methodology that could be applied to cropping systems (Litrico and Violle, 2015; Martin and Isaac, 2015; Wood et al., 2015; Damour et al., 2018). However, trait-based approach requires extensive data collection to develop trait databases for crop species or varieties and document relationships between traits and services (Barot et al., 2017). As a neglected group of species, pulses are particularly difficult to document (Barot et al., 2017), for implementing a trait-based approach.

In a previous study, Guiguitant et al. (2020) set up a database suitable for functional trait approach on pulses. The database gathered disparate data from multiple published and/or public sources and contained data on plant functional traits for 43 pulse species, and agroecosystem properties recorded at canopy level for a subset of these species. The analysis of the functional trait space highlighted three ecological strategies among cultivated pulses that were similar to those already described in spontaneous species in the leaf-seed-height (LSH) scheme (Westoby, 1998). However, reduced data on agroecosystem properties and data disparity did not allow to identify clear link between traits and agroecosystem properties. In an applied perspective of supporting the integration of pulses in cropping systems, a consolidated analysis of trait–properties relationships is still needed to identify which traits would make legume species more productive, competitive, or adapted to low nitrogen or water conditions and favor production services. Trade-offs between plant traits and how they determine the different strategies also require more attention.

The aim of the present study was to identify pulses traits and/or trait combinations that can be used as predictors of yield and supporting agroecosystem services, further accounting for the relationships between traits and between agroecosystem properties. Original data were collected on 30 pulse varieties from 10 different species monitored in similar environmental conditions. Agroecosystem properties were measured at the canopy level, whereas functional traits, i.e., morphological, physiological, or phenological traits supposed to have direct or indirect effects on agroecosystem properties, were measured at the individual or organ level. We combined these original data collected within uniform environmental conditions with literature data collected in diverse agronomic situations (see Guiguitant et al., 2020) to assess the effect of trait measurement conditions on trait values and whether it could distort the relationships between traits and agroecosystem properties.

## Materials and Methods

### Plant Material and Growing Conditions

A field experiment was conducted at UCOSEM’s experimental farm in Lectoure (lat./long 43.911158/0.666182) on a deep clay-limestone soil. Three varieties for each one of the 10 pulses species (Table 1) were planted in two sequential batches/sub-experiments on contiguous fields of approximately 1 ha each. According to their temperature requirements: Cool season legumes were sown after the last negative temperatures, on March 14, 2018, and warm season legumes were sown on June 7, 2018, when daily temperature was over 15°C. Each batch/sub-experiment was laid out in split plot design with four replicates, varieties being subplots and species the main plot. Plots were 6 m long and counted four rows. Preceding crop was sunflower, and fields were fertilized with 0/22/8 NPK (350 kg ha^–1^) fertilizer before sowing. Each species was manually inoculated before sowing with an adequate strain of rhizobia (provider: UMR LSTM, Montpellier, France). Sowing density was chosen according to agronomical recommendations for each species and ranged from 10 to 150 plants m^–2^. Manual weeding and drip irrigation were performed on a regular basis from sowing. Soil moisture was recorded weekly in each block and for each species at three depths (0–20, 20–40, and 40–60 cm) using TDR probes installed vertically at sowing (mini TRASE^TM^ system with buriable probes, Soilmoisture Equipment Corp., CA) and confirmed that the plants did not experiment any water deficit in none of the two sub-experiments.

**TABLE 1:** List of the 10 species included in the experiment with a description of the three varieties associated to each species, the abbreviation (Abb.) used in the study, sources of the seeds, and the ß value used for %Ndfa computation.

**Species**	**Varieties**	**Abb.**	**Source**	**β (‰)**
*Vigna mungo*	ISRA 58-77 (V1), ISRA 66-41 (V2), ISRA 67-30 (V3)	Vmu	ISRA, Sénégal	−1,75
*Vigna unguiculata*	YACINE (V1), SAM (V2), IT98K-1092-1-1 (V3)	Vun	ISRA, Sénégal	−1,61
*Vigna aconitifolia*	SH_VM (V1), SH_VA (V2), Tvu 45 35 (V2)	Vac	ISRA, Sénégal	−0,91
*Lotus tetragonolobus*	Landrace	Lte	Palmbeach medicinal herb, rareexoticseed, hobbyseed	−0,12
*Phaseolus vulgaris*	Red kidney (V1), zorro (V2), michelet (V3)	Pvu	Epi de Gascogne, France	−2,16
*Cicer arietinum*	Elmo (V1), billy bean (V2), orion (V3)	Car	Epi de Gascogne, France; USDA Pullman, WS	−1,75
*Lens culinaris*	Belezana (V1), Richela (V2), Anicia (V3)	Lcu	Epi de Gascogne, USDA Pullman, WS	−0,56
*Trigonnella foenum-graecum*	FENUSOL, landrace	Tfo	Epi de Gascogne, France; ICARDA, Morroco	−0,42
*Lathyrus sativus*	Wassie (V1), Almenaza (V2), Chicharo (V3)	Lsa	Epi de Gascogne, France	−0,38
*Vicia faba*	Aguadulce (V1), alfia (V2), lobaba (V3)	Vfa	ICARDA, Morroco	−0,5

### Measurements of Individual Plant Traits

Occurrence of phenological stages (date of 50% flowering, date of physiological maturity, i.e., when more than 50% of the pods were yellow-brown) was recorded in calendar days and then converted into thermal units using a base temperature of 0°C for *Cicer arietinum* and *Vicia faba*, 2°C for *Lens culinaris*, 3.5°C for *Lathyrus sativus*, 4.1°C for *Trigonella foenum-graecum* (Covell et al., 1986; Lamichhane et al., 2020), 4.7°C for *Lotus tetragonolobus* (Moot et al., 2000), 6°C for *Phaseolus vulgaris* (López et al., 2003), and 8.5°C for *Vigna* sp. (Craufurd et al., 1996). This allowed to compute degree days from sowing to flowering (FLO) and degree days from sowing to maturity (MAT).

Biomass was harvested at flowering stage and maturity. At each sampling date, plants were collected on 0.5 m^2^ quadrats, in the two central rows to avoid border effect. Plant number was recorded, and then leaves, stems, flowers, and pods were detached and then dried separately at 60°C until constant weight. Leaf and stem dry mass ratio over total aboveground plant mass (LMR and SMR, respectively) was calculated at flowering, and harvest index (HI) was calculated at maturity. Leaf area ratio (LAR; calculated as total plant leaf area (LA) over total aboveground mass), total LA (mm^2^ plant^–1^), specific LA (SLA, cm^2^ g^–1^), and leaflet length (LL, mm) as well as leaflet width (LW, mm) were measured at flowering (mean of the measurement of every leaf of a plant sub-sample), whereas thousand seed weight (TSW, g) was determined at harvest.

Total nitrogen content was determined (Vario PyroCube auto-analyzer and Precision mass spectrometer) on leaves, stems, and grain samples collected at maturity and at flowering. Total plant nitrogen content was computed both at flowering and maturity. Leaf nitrogen content (LNC, mg g^–1^), and seed nitrogen content (SNC, mg g^–1^) were determined at flowering and at maturity, respectively.

Phyllochron was calculated from observations of three tagged plants on each plot for which emerged leaves were counted weekly (cool season legumes) or twice a week (warm season legumes).

The rate of soil coverage was estimated by measuring soil cover weekly, using nadiral photos taken at the same location within each plot, with a large angle camera at chest height, and then analyzed with ImageJ software (Schneider et al., 2012). Soil cover was modeled as a logistic function of thermal time. The maximal rate of soil coverage was defined as the slope at the inflexion point of the logistic curve.

### Measurement of Agroecosystem Properties

Agroecosystem properties were measured on the same experimental plots (Table 2). Grain yield (GY, t ha^–1^), biomass yield (BY, t ha^–1^), and biomass measured at flowering (t ha^–1^) were determined from plant sampling on 0.5 m^2^ quadrats. Water use efficiency (WUE) was computed as grain mass produced per mm of water input (rainfall and irrigation). Maximum soil cover (%), LA index (LAI), and the duration (in thermal units) of the phase when soil coverage is above 80% of the maximum (soil cover duration) were recorded. Finally, the capacity to fix nitrogen from the atmosphere was estimated through the percent of nitrogen derived from biological fixation at flowering and at maturity, measured with the natural isotopic dilution method, using barley and maize grown in the experiment outer borders as control plants for cool season and warm season legumes, respectively. From the ^15^N abundance (d^15^N) of the samples, the proportion of plant N derived from atmosphere (%Ndfa) was calculated from Eq. 1 (Shearer and Kohl, 1986):

**TABLE 2:** List of agroecosystem properties and traits measured in the experiment with their respective abbreviation and unit.

**Variable**	**Abbreviation**	**Unit**
***Agroecosystem property***		
Grain yield	GY	t ha^–1^
Biomass yield	BY	t ha^–1^
Leaf area index	LAI	
% Nitrogen derived from atmosphere at maturity	Ndfa_MAT	%
% Nitrogen derived from atmosphere at flowering	Ndfa_FLO	%
Water use efficiency	WUE	kg ha^–1^ mm^–1^
Biomass at flowering	B_FLO	t ha^–1^
Maximal soil cover	Maximum soil cover	%
Soil cover duration	Soil cover duration	Thermal unit
***Traits***		
Leaf area	LA	mm^2^
Specific leaf area	SLA	cm^2^ g^–1^
Leaflet length	LL	mm
Leaflet width	LW	mm
Leaf nitrogen content	LNC	mg g^–1^
Plant N content at flowering	Nplant_FLO	g plant^–1^
Plant N content at maturity	Nplant_MAT	g plant^–1^
Phyllochron	Phyllochron	Thermal unit
Soil cover rate	Cover rate	
Degree days to maturity	MAT	Thermal unit
Degree days to flowering	FLO	Thermal unit
Thousand seed weighed	TSW	G
Leaf area ratio	LAR	–
Leaf mass ratio	LMR	–
Stem mass ratio	SMR	–
Harvest index	HI	–
Seed nitrogen content	SNC	mg g^–1^

(1) $$\%\mathrm{Ndfa}=100\frac{\delta \,15\,N_{\mathrm{ref}}-\delta \,15\,N_{\mathrm{legume}}}{\delta \,15\,N_{\mathrm{ref}}^{-\beta }}$$

Where *β* is the ^15^N natural abundance of the N derived from biological nitrogen fixation (BNF) in the aerial tissue of the legume, δ15*N*_*ref*_ is the ^15^N abundance in the control plant (maize or barley) tissues, and δ15*N*_*legume*_ is the ^15^N abundance in legume tissues. The *β* values used are detailed in Table 1 (Unkovich et al., 2008).

### Data Analysis

Data were first analyzed through the prism of the framework presented in Guiguitant et al. (2020) in order to compare the variability observed with the global functional diversity of pulses species. Data of the present field measurements were projected as supplementary individuals in a probabilistic principal component analysis (PPCA) (Tipping and Bishop, 1999) computed with nine common functional traits (DM, DF, HI, LL, LW, LA, SLA, LNC, and TSW) values taken from Guiguitant et al. (2020) database. We used Euclidian distance to quantify the distance between the scores of barycenters of each species as calculated from field data and previously published in Guiguitant et al. (2020). The consistency of species ranking in trait values across literature and experimental data was tested with Spearman’s rank correlation analysis.

Second, a principal component analysis (PCA) was performed on experimental data including all traits measured. Agroecosystem properties were added as supplementary variables, in order to investigate how they fit into the phenotypic space defined by the traits. The appropriate number of PCs was determined with Kaiser’ criterion which selects components that correspond to eigenvalues larger than 1. A between and within-class correspondence analysis was carried out to evaluate the percentage of variance captured by the intra and inter-specific diversity (Doledec, 1987).

Finally, trade-offs among agroecosystem properties were analyzed using a hierarchical clustering analysis (HCA). A classification and regression tree (CART) analysis was done on raw data (*n* = 120) to identify how functional traits could allow to predict the clusters of varieties obtained from the HCA. Regression trees are prediction models obtained through machine-learning algorithms that recursively partition the data space in order to fit the simplest prediction model within each partition. The resulting partitioning can be represented graphically as a decision tree. The random forest aggregation method, which consists in adding a random component to the choice of variables in the model, was also used. This method does not allow to generate a visual decision tree, but it provides indices proportional to the importance of each variable in the aggregated model and thus its participation to the discrimination between groups of individuals (here, varieties). Mean decrease in Gini impurity (MDG) can be defined as the total decrease in node impurity (weighted by the proportion of samples reaching a given node) averaged across all of the trees that make up the forest. The most important variables to the model will have the largest MDG values; conversely, the least important variable will have the smallest MDG values.

All statistical analyses were performed in the computing environment R 3.5.2 (R Core Team, 2018) using *pcaMethods* package (Stacklies et al., 2007), *rpart* package (Therneau et al., 2015), *ade4* package (Dray and Dufour, 2007), and *randomForest* package (Breiman, 2001).

## Results

### Consistency of Pulses Functional Trait Space

A PPCA was performed on the 43-species-database from Guiguitant et al. (2020) using only traits that were also measured in the present field experiment. The first three PC axes of this PPCA explained 70% of total variance observed in the literature (Figure 1). PC1 (34%) was associated with morphological traits, especially leaf traits (LL, LW, LA, and to a lesser extend TSW). PC2 (24%) was positively associated with late flowering (DF) and maturity (DM) and to a lesser extend HI. PC3 (12%) was strongly associated to SLA and LNC. Experimental data variability on PC axes was in the same range as the literature variability. Experimental variance was close to 100 and 60% of literature variance on PC1 and PC2, respectively. On the contrary, the variance of supplementary individuals on the third axis (driven by SLA and LNC) was almost three times higher than the variance of active data from the literature (Appendix 1). For all other traits, field measurements range was always narrower than the one observed in Guiguitant et al. (2020), for a larger set of species (Appendix 1).

**FIGURE 1:**
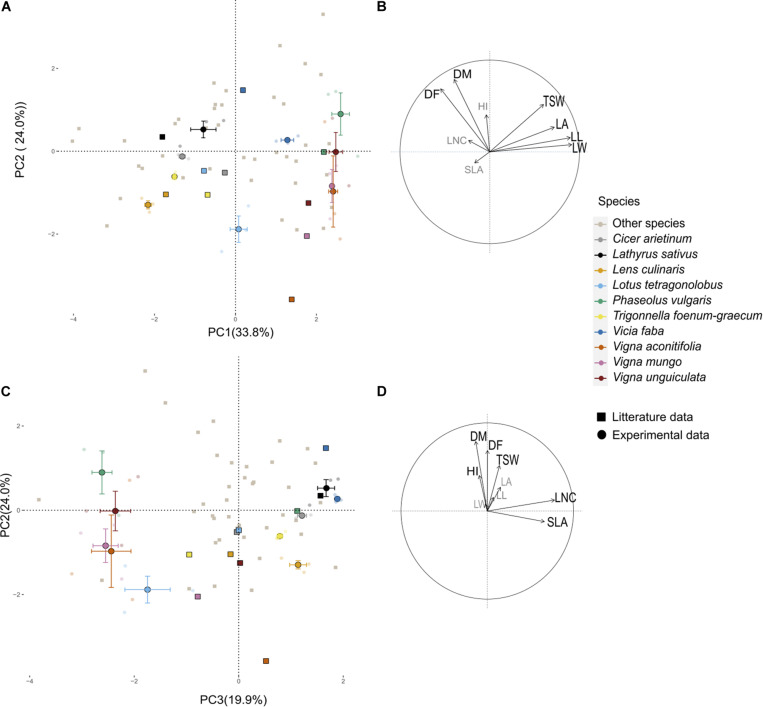
Principal component analysis (PCA) performed on nine functional traits collected on 43 pulses species. **(A,C)** Individual species projection on the first three axes of the PCA; light gray points represent the 43 species from literature (Guiguitant et al., 2020) and colored points are from the current experimental study projected as supplementary individuals. Transparent points are the data of the four plots for the three varieties of the 10 species, the larger opaque points represent the barycenter of each species, and the horizontal and vertical bars represent the standard deviation along axes. Visualization of the variables and correlation circles on **(B)** PC1–PC2 and **(D)** PC2–PC3 planes. See Table 2 for abbreviations of traits.

Distance between the barycenters of each species as tested in the present experiment and from the literature database was short in the first plane of the PPCA (Figure 1). Species ranking in literature versus present data remained conserved on PC1 (ρ = 0.85, *p* = 0.003) and to a lesser extent on PC2 (ρ = 0.53, *p* = 0.10). Species rankings were rather inconsistent on PC3 (ρ = 0.24, *p* = 0.12). Cool season species had barycenters closely positioned along the three dimensions of the PPCA (Euclidian distance lower than 2; Table 3). Among them, *C. arietinum* and *T. foenum-graecum* had the highest Euclidian distance mostly because of the divergence with literature on the third axis, while *V. faba* diverged mostly on the second axis. For warm season species, distances between barycenters from the literature and from the present experiment were larger, on PC3 but also on PC2 for *Vigna* sp.

**TABLE 3:** Euclidian distance between literature and current study coordinates of the barycenter of each species on the firsts three PCA axes.

**Species**	**PC1**	**PC2**	**PC3**	**Three PCs**
*Cicer arietinum*	1.1	0.39	1.25	1.68
*Lathyrus sativus*	1.0	0.18	0.11	1.03
*Lens culinaris*	0.4	0.25	1.29	1.39
*Lotus tetragonolobus*	0.9	1.41	1.74	2.40
*Phaseolus vulgaris*	0.4	0.92	3.74	3.87
*Trigonella foenum-graecum*	0.8	0.44	1.73	1.97
*Vicia faba*	1.1	1.20	0.22	1.64
*Vigna aconitifolia*	1.0	2.61	2.96	4.08
*Vigna mungo*	0.6	1.21	1.76	2.23
*Vigna unguiculata*	0.7	1.23	2.39	2.77

### Characterization of Functional Trait Diversity

**FIGURE 2:**
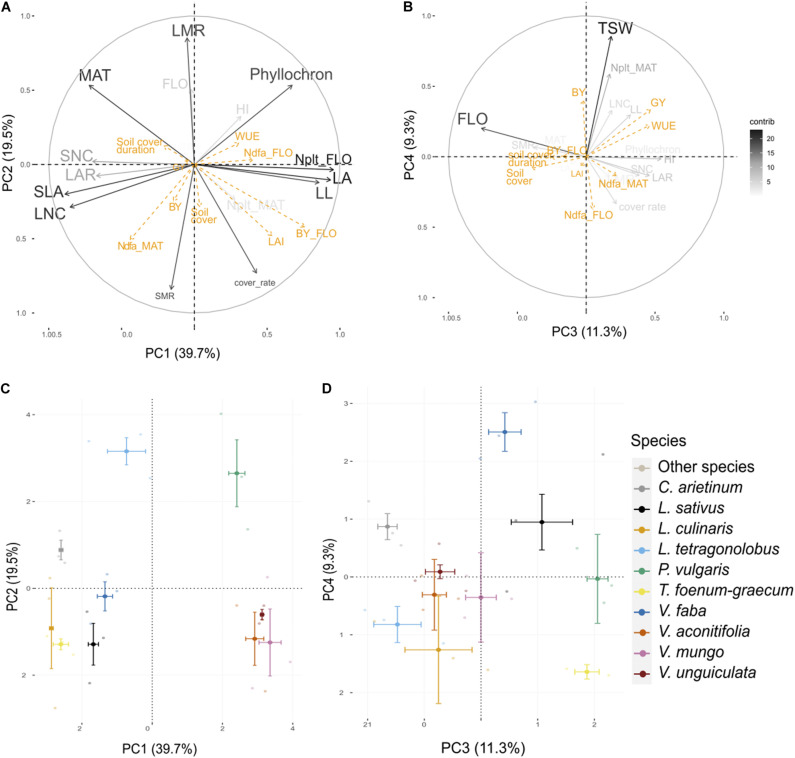
Principal component analysis (PCA) performed on traits measured on three varieties of 10 species. Visualization of the variables and correlation circles on **(A,C)** PC1–PC2 and **(B,D)** PC3–PC4 planes. Gray scale represents the contribution of each variable to axes construction. Orange variables are agroecosystem properties, used as supplementary variables. See Table 2 for abbreviations of traits and properties. Soil cover stands for maximal soil cover.

The first four axes of the PCA performed on 30 individuals and 16 traits from the present experiment (Table 2) captured 80% of the total variance (Figure 2). Interspecies variance explained 81% of the total variance, while intra-species explained 19%.

Leaf attributes were strongly associated with PC1 (40%). On the first principal component (PC1), LA, LL, and plant N content at flowering were negatively correlated with SLA, LNC, SNC, and LAR. This axis discriminated cool season species which were characterized by small leaflets and small LA with high leaf and seed nitrogen content and a large LAR, from warm season species characterized by large leaflet, high LA, and high plant nitrogen content. PC2 (20%) was positively correlated to LMR and negatively correlated to SMR and cover rate. The cover rate was significantly negatively correlated to MAT (ρ = –0.72, *p* < 0.001). SMR was also significantly negatively correlated to the phyllochron (ρ = –0.60, *p* < 0.001). *L. tetragonolobus* and *P. vulgaris* were apart from all other species on PC2, and both expressed a high LMR at flowering. PC3 (11%) and PC4 (9%) were associated to FLO and TSW, respectively. PC3 separated *V. faba* from *L. culinaris* and *L. tetragonolobus*, whereas PC4 discriminated *C. arietinum* and *P. vulgaris.*

All agroecosystem properties, except LAI, biomass at flowering and Ndfa at maturity were not well represented in the factor planes formed by the fourth PCs constructed with traits (Figure 2). LAI and biomass at flowering were relatively well represented in the first plane and were significantly positively correlated to LL (ρ = 0.46, *p* < 0.001; ρ = 0.63, *p* = 0.01, respectively) and LA (ρ = 0.67, *p* < 0.001; ρ = 0.83, *p* < 0.001, respectively) as well as cover rate (ρ = 0.59, *p* < 0.001; ρ = 0.60, *p* < 0.001, respectively). Although Ndfa at flowering was poorly represented on the first PC, this property was significantly positively correlated to plant N content at flowering (ρ = 0.41, *p* = 0.02). Ndfa at maturity was not correlated to Ndfa at flowering (ρ = 0.04, *p* = 0.83) but was significantly positively correlated to LNC (*r* = 0.49, *p* = 0.005) and SLA (ρ = 0.57, *p* = 0.001) as well as with SMR (ρ = 0.43, *p* = 0.02). The soil cover duration was inversely correlated to LAI (ρ = –0.21, *p* = 0.0428) and biomass at flowering (ρ = –0.22, *p* = 0.033) and significantly positively correlated with FLO (ρ = 0.47, *p* = 0.009) and to a lesser extent with MAT (ρ = 0.33, *p* = 0.08). Maximum soil cover was not correlated to soil cover duration (ρ = –0.03, *p* = 0.86) and was slightly associated with SMR (ρ = 0.35, *p* = 0.05). Finally, GY and WUE were positively correlated to PC3 which was mostly determined by FLO, whereas BY, and to a lesser extent GY and WUE, were positively associated to PC4 alongside TSW.

### Trade-Offs Between Agroecosystem Properties

**FIGURE 3:**
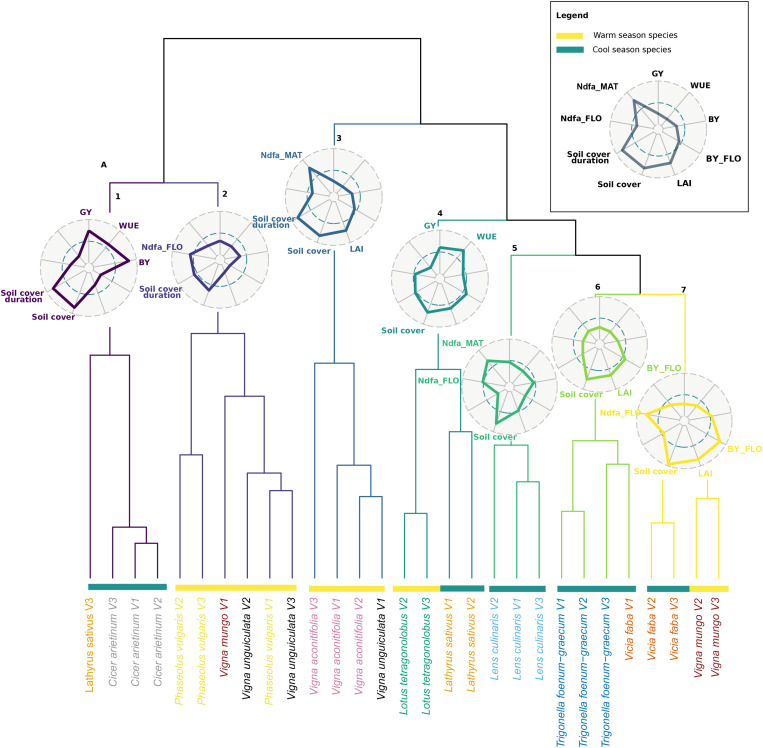
Hierarchical clustering analysis (HCA) of the 30 pulse varieties based on mean value of agroecosystem properties (*n* = 3). The names of varieties belonging to the same species are written in the same color. Warm and cool season species are depicted with yellow and green bars, respectively. Radars represent the mean (standardized to maximum) value of agroecosystem properties of each cluster. Colors in radar plots correspond to branch color in the HCA.

The classification analysis revealed seven clusters of species and varieties based on their agroecosystem properties (Figure 3). All varieties of a species generally grouped into the same cluster. *V. aconitifolia* varieties were all in cluster 3 together with one variety of *Vigna unguiculata*. This cluster is apart from a group of close clusters composed essentially by cool season species; *L. sativus* varieties were grouped with *L. tetragonolobus* (cluster 4), *L. culinaris* was alone in cluster 5, cluster 6 was dominantly occupied by *T. foenum-graecum*, and cluster 7 grouped both *V. faba* and *Vigna mungo*. These groups were distant from cluster 1 essentially represented by *C. arietinum* and cluster 2 that contained varieties of *P. vulgaris*, and of *V. unguiculata*.

Each cluster was characterized by a combination of values of agroecosystem properties. Cluster 1 grouped species with high production (GY and BY) as well as a high maximum soil cover and soil cover duration, but a low Ndfa and a low biomass and LAI at flowering. Cluster 2 was characterized by a low biomass at flowering as well as a low LAI at flowering and maximum soil cover but a high Ndfa at flowering and a long soil cover duration. Cluster 3 varieties expressed the lowest yield as well as the lowest WUE but high Ndfa at maturity, a high LAI at flowering, and a long cover duration. Cluster 4 varieties expressed the highest WUE concomitantly with a high GY and a low Ndfa at both stages. Varieties of cluster 5 expressed high Ndfa both at flowering and maturity and high maximum soil cover. Yet, they showed a low maximum soil cover and soil cover duration. Cluster 6 was characterized by a low BY and GY, low Ndfa both at flowering and maturity, and poor soil covering capacity, yet LAI and soil cover at flowering were high. Finally, cluster 7 was composed of varieties producing the highest biomass at flowering, along with the highest maximum soil cover, LAI, and Ndfa at flowering.

### Relevant Traits for Prediction of Agroecosystem Properties and Structuration of Functional Diversity

**FIGURE 4:**
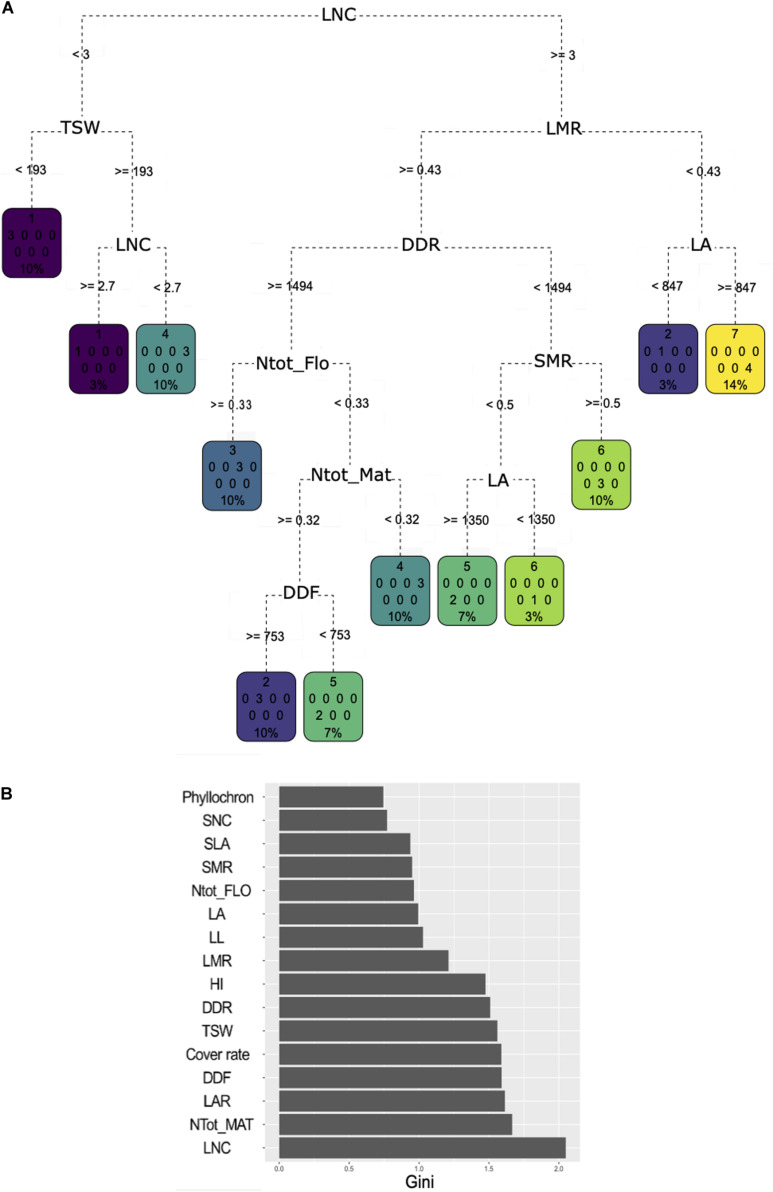
**(A)** Classification and regression tree (CART) regression for prediction of membership to one of the seven clusters based a hierarchical clustering analysis of the 30 pulse varieties based on agroecosystem properties (see Figure 3). Root nodes represent single input variables (traits) and related split point used to make the prediction; the best discriminant is chosen at each step of the classification procedure. Leaf nodes contain mean, number, and percentage of observations of the predicted variable (cluster membership). **(B)** Gini decrease value for each trait obtained through a random forest procedure. A high Gini decrease value means the variable considered allowed to gain purity in the process of classification for most of the nodes in the CART it was associated with during the random forest procedure.

Classification and regression tree analysis showed very diverse trait combinations to predict the cluster affiliation (Figure 4A). LNC was identified as the most important trait to discriminate the clusters as it had the highest MDG value, which means that it allowed to gain purity in the process of classification for most of the nodes in the CART it was associated with (Figure 4B). LNC was assigned to the first division node and allowed to sort cluster 1 varieties apart from the remaining ones. Cluster 1 varieties have an LNC between 2.7 and 3 mg N g^–1^. Varieties of cluster 4 were separated. Half of them fell on the same side of the tree as cluster 1 but exhibited lower LNC value (below 2.7 mg N g^–1^), while the other half had an average LNC above 3 mg N g^–1^. Varieties in this second cluster 4 half also had an LMR above 43% concomitantly with low plant N content at flowering and at maturity and a longer cycle. LMR, DDR, and plant N content at maturity had strong MDG unlike the plant N content at flowering. Varieties with an LMR under 0.43 and having high MAT were assigned either to cluster 3 (characterized by a high plant N content but only at flowering), or cluster 2 or 5 (both characterized by high plant N content at flowering and maturity). Varieties in cluster 2 flowered later than those in cluster 5. Short cycle varieties were dominantly associated to cluster 6 but were also found in cluster 5 if they also expressed a high LA. Finally, cluster 7 was characterized by an LMR under 43%.

Relying on MDG, plant N content at maturity was the second most important trait to discriminate the clusters. Phyllochron, SNC, SLA, and LL were not selected in the tree and had very low mean decrease in Gini value. LAR was not used in the trees, but had a higher mean decrease in Gini value than LA and LMR. Yet, LA and LMR captured more variability in the PCA than LAR. Finally, cover rate did not appear on the tree despite its high mean decrease in Gini value, probably due to its negative correlation with DDR.

## Discussion

### Traits Related to Resource Acquisition Are More Plastic Across Growing Conditions and Varieties Than Architectural Traits

Through functional traits, researchers usually seek to make generalizable predictions across organizational and spatial scales (Adler et al., 2013) and therefore usually use data collected in diverse environment and management contexts. On the contrary, field measurements sharing similar environment as in the present study deliver a local picture of functional diversity which variations can be solely attributed to genotypic determinants. Several studies have demonstrated that despite the significant plasticity of most plant traits across environments, the classification of species for a given trait remains fairly consistent in space and time (Garnier et al., 2001; Kazakou et al., 2014; Tribouillois et al., 2015). Yet, other studies have reported a significant effect of plasticity on species ranking across environments, particularly with respect to traits associated with light acquisition and nitrogen nutrition (Lipowsky et al., 2015; Siebenkäs et al., 2015; Keenan and Niinemets, 2017). These inconsistencies may impair the use of simple aggregation functions (means, medians, etc.) for generalized trait-based approaches at the species level.

In the present study, we compared common field measurements to the meta-analysis by Guiguitant et al. (2020) to analyze how environmental and management conditions could alter trait values and distort their relationships. When using the experimental dataset, where all data were acquired in a common environment, species rankings on the PC axis summarizing the variation of architectural traits were fairly consistent with literature ranking. However, rankings on the axis summarizing SLA and LNC variation diverged between the two datasets. This contrast mainly concerned warm-season species, particularly *Vigna* sp. for which divergence between the two datasets was also noted in terms of phenology. Number of days required to reach maturity was generally lower in literature than in experimental data. This comparison between local measurements and literature averages suggests that some traits are more plastic than others and therefore contribute differently to frame legumes diversity depending on the conditions of observation.

### Intraspecific Variation Contributed Only Marginally to Global Variability

Results from the PCA showed that the total trait variance was mainly explained by the variance between species. Variation in intraspecific traits is generally considered of marginal importance compared to differences in interspecific traits (McGill et al., 2006; Hulshof and Swenson, 2010). However, several recent studies have shown that intraspecific variation in traits accounts for a substantial proportion of overall plant community variability (Jung et al., 2010; Albert et al., 2011; Roscher et al., 2015; Siefert et al., 2015). The small number of varieties considered in this study may have not been able to highlight such diversity.

### Leaf Morphological Traits Confirmed as Variability Drivers Among Pulses

Guiguitant et al. (2020) found that seed and leaf traits exhibited the greatest variability among 43 pulse species. In the present study, leaf traits (LA and LL) remained an important variability driver that was correlated to the first PCA axis, whereas seed size, associated to the first axis in the literature analysis, poorly contributed to the total variance across varieties (associated only with the fourth PCA axis) and was decoupled from leaf size, which is consistent with LHS scheme (Westoby, 1998).

The experimental data confirmed that LL is an important determinant of legume diversity. This trait was strongly related to LNC and to a lesser extent SLA. This finding can be related to the well know leaf economic spectrum (LES; Wright et al., 2004). The LES reflects a trade-off between a leaf’s lifespan and its maximum net photosynthetic rate. Yet, relations between LNC and photosynthesis in grain legumes have been questioned (Adams et al., 2016). Thus, the fact that this axis reflects a gradient in leaf photosynthetic capacity is uncertain. However, a high LNC could have other biological implications such as remobilization to the reproductive organs (Davies et al., 2000; Schiltz et al., 2005). Indeed, SNC was correlated with LNC in our experiment. Small leaflet associated with a high LNC could support the growth of N-rich legume seeds through remobilization.

### Yield Is Inversely Related With Ndfa and Soil Cover

Nitrogen fixation by legumes is generally expected to contribute to improving soil fertility through the mineralization of crop residues such as senescent leaves or stems, or roots. In the current study, Ndfa at flowering and Ndfa at harvest in grain tissue varied almost independently. Clustering reveled three types of “high fixing species”: cluster 7, cluster 3, and cluster 5. On the other hand, cluster 1 or 6 expressed a low N fixation capacity. “High yielding” species were clustered in cluster 1 and, to a lesser extent, cluster 4 both concerned by low fixation. Cluster 5 fixed a lot of N during flowering and produced a decent yield. However, yield in cluster 5 was lower than that of clusters 1 or 4. Conversely, the very low yielding species (cluster 3) expressed a significant increase in relative Ndfa between flowering and maturity. Other multiple species comparisons confirmed the negative relation between BNF and seed yield (Liu et al., 2019). Some studies reported BNF over a certain level results in a reduction of yield (Mesfin et al., 2020).

The multi-faceted benefits of cover crops are well known (Blanco-Canqui et al., 2015), but land cover is often not considered for cash crops and the interaction between cover capacity and yield is rarely discussed. Maximum cover and cover duration appeared to be related to biomass at flowering and LAI as well as to yield properties. Cluster 7 expressed an important cover at flowering as a result of an important biomass and a large LA. However, cluster 1 and 3 had a low biomass at flowering and a relatively high maximum cover that lasts long, probably because of a delayed senescence. Except for cluster 1, most of those species had low yield. Delayed senescence, by prolonging photosynthesis, is usually expected to contribute to increasing the yield of high-carbon crops such as cereals (Gregersen et al., 2013). However, studies suggest that the stay-green trait may have limited value for high N species (Ismail et al., 2000; Kumudini, 2002) as its value lies in an improvement of remobilization capacity. This corroborates the absence of relation we found between yield and duration of ground cover.

### Trait-Based Predictions of Agroecosystem Properties

Understanding the links between functional traits and agroecosystem functioning is fundamental for optimal management of agroecosystem services (Zhang et al., 2007; Litrico and Violle, 2015; Wood et al., 2015). As noted earlier, leaf characteristics and especially LNC were crucial in describing pulse diversity. Other traits such as LAR, total nitrogen, seed size, and cycle length are also important, which is consistent with the study of Guiguitant et al. (2020). The decision tree did not highlight all these traits but discriminated specific functional characteristics for each cluster. Clusters 1 and 4 (high yielding species) were characterized by low LNC values which is consistent with their low N fixation. Cluster 7 (high biomass at flowering) was characterized by a large LA but a low biomass investment in leaves. Clusters 6 and 5 (medium maximum soil cover and low value for every other properties) were characterized by a short cycle, with cluster 5 (high nitrogen fixation) having a higher LA. Clusters 2 (medium value for every properties) and 3 (high N fixation at maturity and long cover duration) were characterized by a long cycle and high nitrogen content at maturity or flowering, respectively.

To conclude on the highlighted linkages, a high LNC value does not translate into higher yields but is generally associated with a high N fixation. Investment in LA is important for N_2_ fixation at flowering but is associated with rapid senescence and low yield. Biomass investment in leaves and a short cycle coincide with low values for almost every property except maximum soil cover. Finally, long cycle and high N content at maturity are related to delayed senescence and maintenance of N_2_ fixation along pod filling.

## Conclusion

Integrating pulses in resilient and sustainable production systems requires a better understanding of their functional diversity and how this functional diversity scales up to provide specific sets of agroecosystem services. Previous studies demonstrated the usefulness of trait value aggregation at the species-level to implement trait-based approaches for a better knowledge of the plant-agroecosystem functioning relationships and increasing our predictive capacity (Lavorel and Garnier, 2002; Violle et al., 2007; Garnier and Navas, 2012; Kazakou et al., 2014; Martin and Isaac, 2015; Wood et al., 2015; Guiguitant et al., 2020). Our experimental results highlighted trade-offs and synergies between agroecosystem properties supported by different pulses species. We were able to demonstrate the relevance of LNC, LAR, and phenology to predict most of the trade-offs observed for agroecosystem properties in those conditions.

However, the present study advocates further for a systematic characterization of the functional diversity (and its plasticity), including intra-specific variability. We argue that the trait-based approach may be easier to implement in crop species where varieties origin and genetics are often better described and accessible, compared with the huge genetic variation encountered in populations of spontaneous species. Application of such trait-based approach would rapidly benefit the selection of species/varieties for specific targeted agroecosystem services provisioning under specific (environmental or management) conditions.

## Data Availability Statement

The original contributions presented in the study are included in the article/supplementary material. Further inquiries can be directed to the corresponding author/s.

## Author Contributions

All authors conceived the ideas and approach together. JG conducted the experiments and data analyses and led the writing of the manuscript. DV and HM contributed substantially to the manuscript through extensive revision. HM designed the experiment. DV participated to data analyses.

## Conflict of Interest

The authors declare that the research was conducted in the absence of any commercial or financial relationships that could be construed as a potential conflict of interest.

## Figures and Tables

**FIGURE 1:**
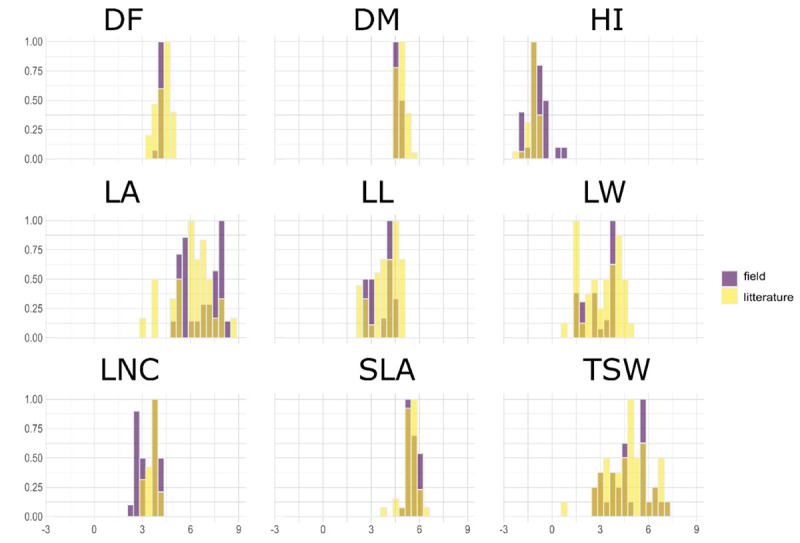
Comparison of traits range for data measured in the present field experiment over 30 varieties issued from 10 species of pulses (violet) and species-level data collected in literature in Guiguitant et al. (2020) for 43 species of pulses (yellow).
